# Stabilization of a chaotic oscillator via a class of integral controllers under input saturation

**DOI:** 10.1038/s41598-023-33201-3

**Published:** 2023-04-12

**Authors:** Ricardo Aguilar-López, Juan L. Mata-Machuca

**Affiliations:** 1 Department of Biotechnology and Bioengineering, CINVESTAV, Mexico City, 07360 Mexico; 2 UPIITA, Department of Advanced Technologies, Instituto Politecnico Nacional, Mexico City, 07340 Mexico

**Keywords:** Applied mathematics, Statistical physics, thermodynamics and nonlinear dynamics

## Abstract

This work presents the straightforward design of an integral controller with an anti-windup structure to prevent undesirable behavior when actuator saturation is considered, and the proposed controller improves the performance of the closed-loop dynamics of a class of nonlinear oscillators. The proposed integral controller has an adaptive control gain, which includes the absolute value of the named control error to turn off the integral action when it is saturated. Closed-loop stability analysis is performed under the Lyapunov theory framework, where it can be concluded that the system behaves in an asymptotically stable way. The proposed methodology is successfully applied to a Rikitake-type oscillator, considering a single input-single output (SISO) structure for regulation and tracking trajectory purposes. For comparison, an equivalent fixed gain integral controller is also implemented to analyze the corresponding anti-windup properties of the proposed control structure. Numerical experiments are conducted, showing the superior performance of the proposed controller.

## Introduction

The control of nonlinear systems with highly complex behavior is currently an important issue in science and engineering^1–4^. As is well known, nonlinear systems present steady-state multiplicity, where unstable homoclinic and heteroclinic manifolds are possible^5,6^ and the local presence of zero eigenvalues in equilibrium points^7,8^, the input multiplicity phenomena, and so on^9,10^ can affect the controllability properties of a specific nonlinear system, complicating the correct design of control laws^11–13^.

The control of nonlinear systems or even the control of chaotic dynamical systems has been studied for several years^14–18^. Controlling chaos via adaptive, sliding-mode, predictive, input-to-state linearizing, fuzzy-logic, neural network, and robust proportional-integral (PI) controllers, among other approaches, has been successfully published in the open literature^19–25^. However, most of the abovementioned control designs are based on complex mathematical frameworks and need to be coupled, for example, with sophisticated optimization algorithms and nonlinear models of systems, which can complicate their real-time application and operational adjustment by engineers^25^. In addition, several other issues remain, one of which is related to the physical restrictions of the chaotic oscillators and the respective manipulable control inputs, as it is well known that the corresponding state variables of oscillators can belong to a compact set that is upper-lower bounded and that the manipulable control inputs also belong to intervals with a minimum and maximum physical value^26–28^.

From the above, a traditional control problem arises, which is the saturation of the control actions. The significance of taking control input saturation into consideration in the design of practical control systems has been well studied. The saturation of a controller diminishes the anticipated closed-loop performance of the system’s dynamics and, in extreme condition, may lead to closed-loop instability^29^.

Now, the analysis of the saturation of control has been performed by anti-windup designs, where the applications to linear systems and PI controllers have been dominant in the open literature^30–33^. PI controllers are widely employed in a vast majority of linear and nonlinear systems, and the proportional term acts to stabilize the dynamic behavior of the system close to the required reference or set point, but high proportional gain values are needed to diminish the offset^34^, i.e., the difference in the current value of the controlled variable and the set point, making the control action very sensible. In addition, proportional controllers are sensitive to noisy measurements, and if the system reaches the set point, proportional control is turned off and the system is in open-loop operation; in this case, if an external disturbance is present, the system can become unstable^34^. To improve the performance of a proportional controller, an integral term of the control error can be added; the integral term is able to eliminate the offset, keep the controller turned on and reject some external disturbances^35^. From the abovementioned information, only the integral term of the linear controllers has been considered to regulate several systems.

Indeed, the saturation of the actuators from the focus of linear controllers has been analyzed by integral windup phenomena, integrator windup or reset windup, which refers to the situation in a proportional integral (PI) feedback regulator, where a large change in set point occurs and the integral term accumulates a significant error as it increases; therefore, the controller is overran and continues to increase as this accumulated error is unwound.

The abovementioned physical restrictions have important impacts on the control designs with an integral term of the PI controller, such that if the controller reaches a saturation condition without reaching the required reference point or trajectory, the whole system in closed-loop operation is considered to be under the named windup condition, whereas the integral part of the controller continues to theoretically add control effort, but it is physically saturated and the ideal affair is physically insolvable due to process saturation; i.e., the output of the process is limited at the bottom or top of its physical scale, making the control error constant, where the specific problem is the redundant overshooting^35^.

Furthermore, the analysis of saturation in terms of control has been performed by anti-windup designs, where the applications to linear systems have been dominant in the open literature^36–39^. Anti-windup designs can involve the controllers being turned off for ranks of time until a response falls back into a satisfactory range, which occurs when the regulator’s process can no longer affect the controlled variable. In practical applications, this task is manually done by process engineers.

This problem can be addressed by initializing the integral regulator to a preset value according to, the value before the problem by adding a set point in a suitable range to disable the integral function until the process variable that needs to be controlled enters the controllable region. This prevents the integral term from accumulating above or below predetermined bounds, and the integral term is back-calculated to constrain the process within the doable bounds. The integral term must be forced to zero every time the control error crosses or is equal to zero. This eliminates the need for the regulator to drive the system to have the same error integral in the opposite direction as the disturbance^40^.

The anti-windup control designs for nonlinear systems are currently a real challenge due to the practical need to design realizable controllers, for example, linearizing controllers via plant inversion; however, this approach is based on a predictive phenomenological model, which is a drawback, as well as optimal control techniques based on Pontryagin’s maximum principle or the Euler-Lagrange approach with important applications, such as secure data transmission and the stabilization of chemical systems via chaotic oscillators^41–43^. For the above reasons, linear PI controllers have been successfully considered, and several approaches have been designed to avoid windup phenomena^44–48^ by turning off the integral part of the controllers for different algorithms; however, these controllers have complex structures, and their physical implementation is difficult.

In this work, a simple control strategy is proposed that only considers an integral of the control error with an adaptive gain, which automatically turns off the control action when the controller is under saturation, avoiding the windup phenomena. The proposed controller is successfully applied to a class of nonlinear chaotic oscillators for regulation and tracking trajectory purposes.

## Chaotic oscillator model

Nonlinear oscillator models have been employed as a benchmark for synchronization purposes under the framework of secure data transmission, and practical examples can be found in Chen, Van der Pool, Rikitake and other works on nonlinear chaotic oscillator models.

The Rikitake chaotic dynamical system is a model that attempts to explain the irregular polarity switching of the Earth’s geomagnetic field^49,50^. The frequent and irregular reversals of the Earth’s magnetic field inspired several early studies involving electrical currents within the Earth’s molten core. One of the first such models to report reversals was the Rikitake-type two-disk dynamo model^51^. The system exhibits Lorenz-type chaos and orbits around two unstable fixed points. This system describes the currents of two coupled dynamo disks.

The 3-D dynamics of the Rikitake-type dynamo system are described as follows:

1 $$\begin{aligned} \begin{array}{l} {\dot{x}}_1=\ x_2-x_1 \\ {\dot{x}}_2=x_1x_3-x_2 \\ {\dot{x}}_3=\gamma ^2\left( 1-x_1x_2\right) -\delta x_3+u \end{array} \end{aligned}$$

They can also be described in vector form:

2 $$\begin{aligned} \dot{{\bf {x}}}={\bf {A}}{} {\bf {x}}+{\bf {f}}\left( {\bf {x}}\right) +{\bf {B}}u \end{aligned}$$

where $${\bf {x}}=\left[ \begin{matrix}x_1\\ x_2\\ x_3\\ \end{matrix}\right] ,$$ $${\bf {A}}=\left[ \begin{matrix}-1&{}1&{}0\\ 0&{}-1&{}0\\ 0&{}0&{}-\delta \\ \end{matrix}\right] , $$ $${\bf {f}}\left( {\bf {x}}\right) =\left[ \begin{matrix}0\\ x_1x_3\\ \gamma ^2\left( 1-x_1x_2\right) \\ \end{matrix}\right] ,$$ and $${\bf {B}}=\left[ \begin{matrix}0\\ 0\\ 1\\ \end{matrix}\right] .$$

The parameter values are $\delta =0.01$ and $\gamma =2.0$.

Here, ${\bf {x}}\ \in {\mathbb {R}}^3$ is the state variable vector, which belongs to a compact set $\Phi$ and, is naturally bounded, and ${\bf {f}}({\bf {x}})$ is assumed to be a smooth vector field, where ${\bf {f}}\left( \cdot \right) : {\mathbb {R}}^3\rightarrow {\mathbb {R}}^3$ and $u({\bf {x}})\in{ \mathbb {R}}$.

## Control design

### Proposition 1

The integral controller in (3) stabilizes the dynamic behavior of the system (2) for regulation and tracking trajectory purposes:

3 $$\begin{aligned} u({\bf {x}})=\ k_3\text {abs}(e_3)\int _{0}^{t}{e_3\left( \sigma \right) d\sigma } \end{aligned}$$

### Proof of Proposition 1

Let us define the control error dynamic of system (1) under controller (Eq. 3) as:

4 $$\begin{aligned} \begin{array}{l} {\dot{e}}_1=\ e_2-e_1 \\ {\dot{e}}_2=e_1e_3-e_2 \\ {\dot{e}}_3=\gamma ^2\left( 1-e_1e_2\right) -\delta e_3+\delta x_{3r}+k_3abs(e_3)e_4 \\ {\dot{e}}_4=e_3 \end{array} \end{aligned}$$

Then, Eq. (4) is rewritten in vector notation:

5 $$\begin{aligned} \dot{{\bf {e}}}={\Gamma }({\bf {e}}){\bf {e}}+{\bf {F}}\left( {\bf {e}}\right) +{\Delta } \end{aligned}$$

with: $${\bf {e}}=\left[ \begin{array}{l} e_1 \\ e_2 \\ e_3 \\ e_4 \\ \end{array} \right] , $$ $$\Gamma ({\bf {e}})=-\left[ \begin{array}{llll} 1 &{} -1 &{} 0 &{} 0 \\ 0 &{} 1 &{} 0 &{} 0 \\ 0 &{} 0 &{} \delta &{} -k_3\text {abs}(e_3) \\ 0 &{} 0 &{} -1 &{} 0 \\ \end{array} \right] , $$ $${\bf {F}}({\bf {e}})=\left[ \begin{array}{c} 0 \\ e_1e_3 \\ -\gamma ^2e_1e_2 \\ 0 \\ \end{array} \right] , $$ and $$\Delta =\left[ \begin{array}{l} 0 \\ 0 \\ \gamma ^2+\delta x_{3r} \\ 0 \\ \end{array} \right] . $$

The abovementioned control error is defined as ${\bf {e}}={\bf {x}}-{\bf {x}}_r$, i.e., the difference between the actual values of the state variable vector and the reference vector. The reference vector, ${\bf {x}}_r$ is a constant vector for the regulation case, and it is variable for the tracking case.

By assuming that $0\le \Vert {\bf {e}}\Vert \le {e}_B$; $0\le {e}_B <\infty$, where ${e}_B$ is the finite upper bound of the control error, let us define:

6 $$\begin{aligned} \Gamma ^{*}= & {} \left[ \begin{array}{llll} 1 &{} 1 &{} 0 &{} 0 \\ 0 &{} 1 &{} 0 &{} 0 \\ 0 &{} 0 &{} \delta &{} k_3e_{B} \\ 0 &{} 0 &{} 1 &{} 0 \\ \end{array} \right] \end{aligned}$$

7 $$\begin{aligned} \bar{{\bf {F}}}= & {} \left[ \begin{array}{l} 0 \\ \bar{f}_2 \\ \bar{f}_3 \\ 0 \\ \end{array} \right] \end{aligned}$$

Let us consider the following quadratic form as a Lyapunov function:

8 $$\begin{aligned} V\left( {\bf {e}}\right) ={\bf {e}}^T {\bf {e}} \end{aligned}$$

The corresponding time derivative is defined as:

9 $$\begin{aligned} \dot{V}\left( {\bf {e}}\right) =2{\bf {e}}^T\dot{{\bf {e}}} \end{aligned}$$

Substituting Eq. (5) into Eq. (9) yields:

10 $$\begin{aligned} \dot{V}\left( {\bf {e}}\right) =2{\bf {e}}^T\left( {\Gamma }({\bf {e}}){\bf {e}}+{\bf {F}}({\bf {e}})+{\Delta }\right) \end{aligned}$$

Equation (10) yields:

11 $$\begin{aligned} \dot{V}\left( {\bf {e}}\right) =-2{\bf {e}}^T\Gamma \left( {\bf {e}}\right) {\bf {e}}+2{\bf {e}}^T{\bf {F}}\left( {\bf {e}}\right) +2{\bf {e}}^T\Delta \end{aligned}$$

By applying the Rayleigh inequality to Eq. (11):

12 $$\begin{aligned}{} & {} \lambda _{min}(\Gamma ^*)\Vert {\bf {e}}\Vert ^2\le \Vert {\bf {e}}\Vert _{\Gamma }^2\le \lambda _{max}(\Gamma ^*)\Vert {\bf {e}}\Vert ^2 \end{aligned}$$

13 $$\begin{aligned}{} & {} {{\bf {e}}^T\Gamma ^*{\bf {e}}\le \lambda }_{min}(\Gamma ^*)\Vert {\bf {e}}\Vert ^2 \end{aligned}$$

14 $$\begin{aligned}{} & {} \Vert {\bf {e}}^T{\bf {F}}({\bf {e}})\Vert \le \Vert \bar{{\bf {F}}}\Vert \Vert {\bf {e}}\Vert \end{aligned}$$

15 $$\begin{aligned}{} & {} \Vert {\bf {e}}^T \Delta \Vert \le \Vert \Delta \Vert \Vert {\bf {e}}\Vert \end{aligned}$$

Then, Eq. (12) to Eq. (15) are substituted into equation (11):

16 $$\begin{aligned} \dot{V}\left( {\bf {e}}\right) \le -2\lambda _{min}\left( \Gamma ^*\right) \Vert {\bf {e}}\Vert ^2+2(\Vert \bar{{\bf {F}}}\Vert +\Vert \Delta \Vert )\Vert {\bf {e}}\Vert \end{aligned}$$

where:

17 $$\begin{aligned} \begin{array}{ll} \Vert 2 {\bf {e}}^T({\bf {F}}({\bf {e}})+\Delta )\Vert&\le 2(\Vert \bar{{\bf {F}}}\Vert +\Vert \Delta \Vert )\Vert {\bf {e}}\Vert _{\Gamma ^{*}} \end{array} \end{aligned}$$

In Eq. (17), $\Vert {\bf {e}}\Vert _{\Gamma ^{*}}$ is defined as

18 $$\begin{aligned} \Vert {\bf {e}}\Vert _{\Gamma ^{*}}=\sqrt{\lambda _{max}(\Gamma ^{*})}\Vert {\bf {e}}\Vert \end{aligned}$$

Therefore, from the above, it can be concluded, by the ultimate boundedness, that the regulation error ${\bf {e}}(t)$ is uniformly bounded for any initial condition ${\bf {e}}(t_0)$, such that ${\bf {e}}\left( t\right) =\{{\bf {e}}(t)|\ \Vert {\bf {e}}\Vert \le \mathfrak {R}; \ \mathfrak {R}>0\}$, and finally:

19 $$\begin{aligned} \mathfrak {R}=\dfrac{(\Vert \bar{{\bf {F}}}\Vert +\Vert \Delta \Vert )\sqrt{\lambda _{max}(\Gamma ^{*})}}{\lambda _{min}(\Gamma ^{*})}\sqrt{\dfrac{\lambda _{max}(\Gamma ^{*})}{\lambda _{min}(\Gamma ^{*})}}>0 \end{aligned}$$

## Numerical experiments and results

Numerical simulations were carried out on a personal computer with an Intel Core i7 processor, and the system in Eq. (5) of ordinary differential equations was numerically solved employing the ode23s library of MATLAB$^{ \text {TM}}$, with the corresponding initial conditions $x_{10} = 0.1$, $x_{20} = 0.1$ and $x_{30} = 0.1$, according to McMillen^51^. A single-input single-output control (SISO) configuration is selected for the system. The system is in the open-loop regime from start up until $t = 100$-time units, where controller (Eq. 3) is turned on, and $x_3$ is proposed as the controlled variable. A first set of simulations is performed for regulation purposes, where the selected reference point or set point is $x_{3r} = 1.0$, and a second set of simulations are performed for the tracking case, where system (1) is forced to follow the trajectory described for $x_{3r} = 2.5\ \sin (0.1t + 0.5)$. For both control requirements, that is, regulation and tracking, the control saturations are given by the following lower and upper bounds:

20 $$\begin{aligned} u({\bf {x}})=\left\{ \begin{array}{lll} u_{max} &{} \text {if} &{} u({\bf {x}})=u_{max} \\ u_({\bf {x}}) &{} \text {if} &{} u_{min}<u({\bf {x}})<u_{max} \\ u_{min} &{} \text {if} &{} u({\bf {x}})=u_{min} \end{array} \right. \end{aligned}$$

We set $u_{min}=-10$ and $u_{max}=20$.

For comparison purposes, a similar standard integral controller with a fixed gain is applied as follows:

21 $$\begin{aligned} u_{in}\left( {\bf {x}}\right) =k_1\int _{0}^{t}{e_3\left( \sigma \right) d\sigma } \end{aligned}$$

Here, to achieve the most similar conditions for the operation of controller (Eq. 21) and controller (Eq. 3), the control gain $k_1 = -1.0$ is equal for both control laws.

**Figure 1:**
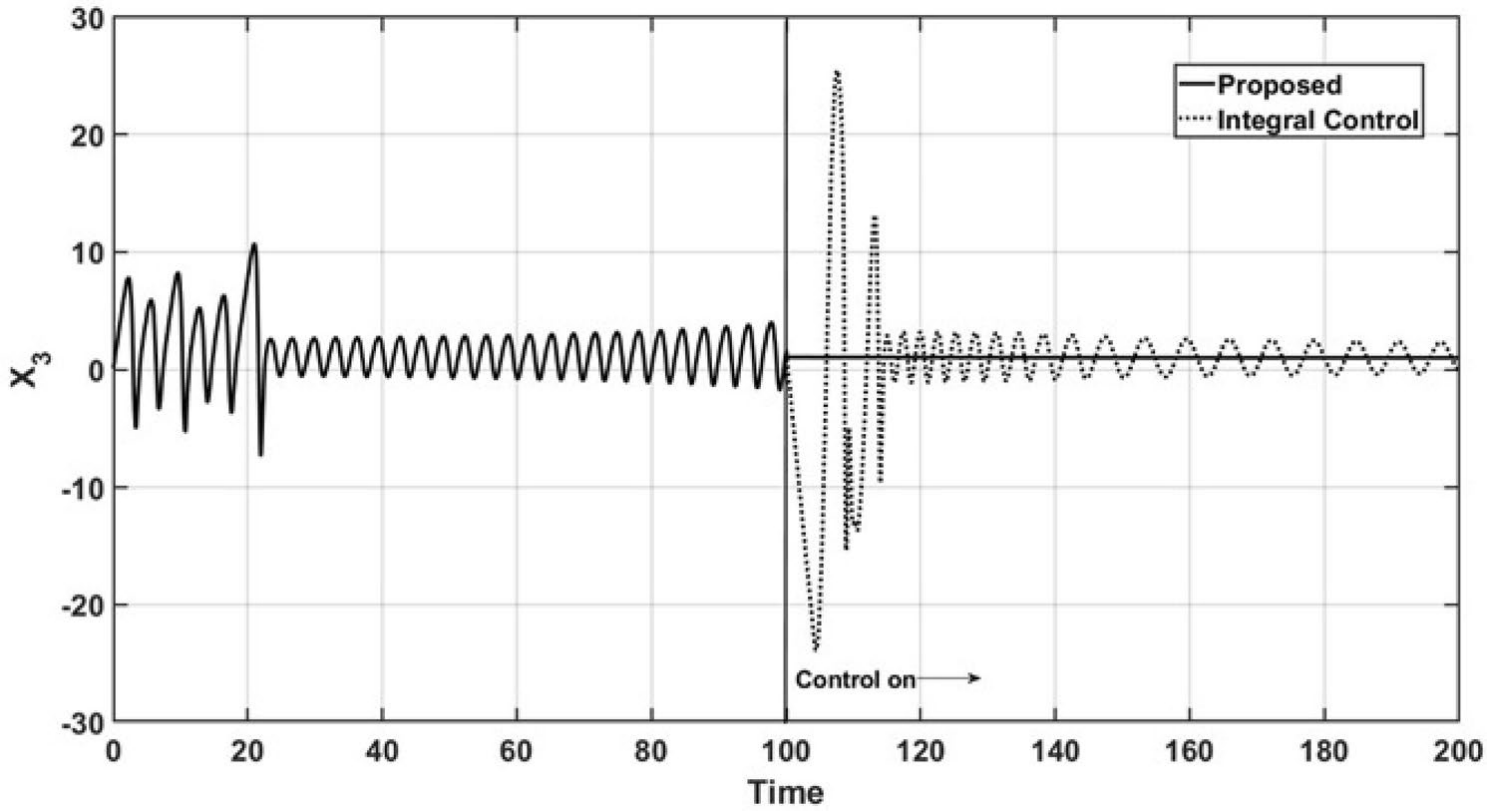
Regulation control of $x_3$.

Figure 1 shows both the open-loop and the closed-loop dynamic behavior of the controlled state variable $x_3$ for the regulation case. As observed, the corresponding trajectory almost immediately reaches the reference point $x_{3r} = 1.0$ for the proposed controller. Additionally, when the integral controller is in operation, the corresponding trajectory has higher oscillatory overshoots and moreover, the integral controller is not able to regulate the dynamic behavior of the controlled state $x_3$, which has a sustained oscillation.

**Figure 2:**
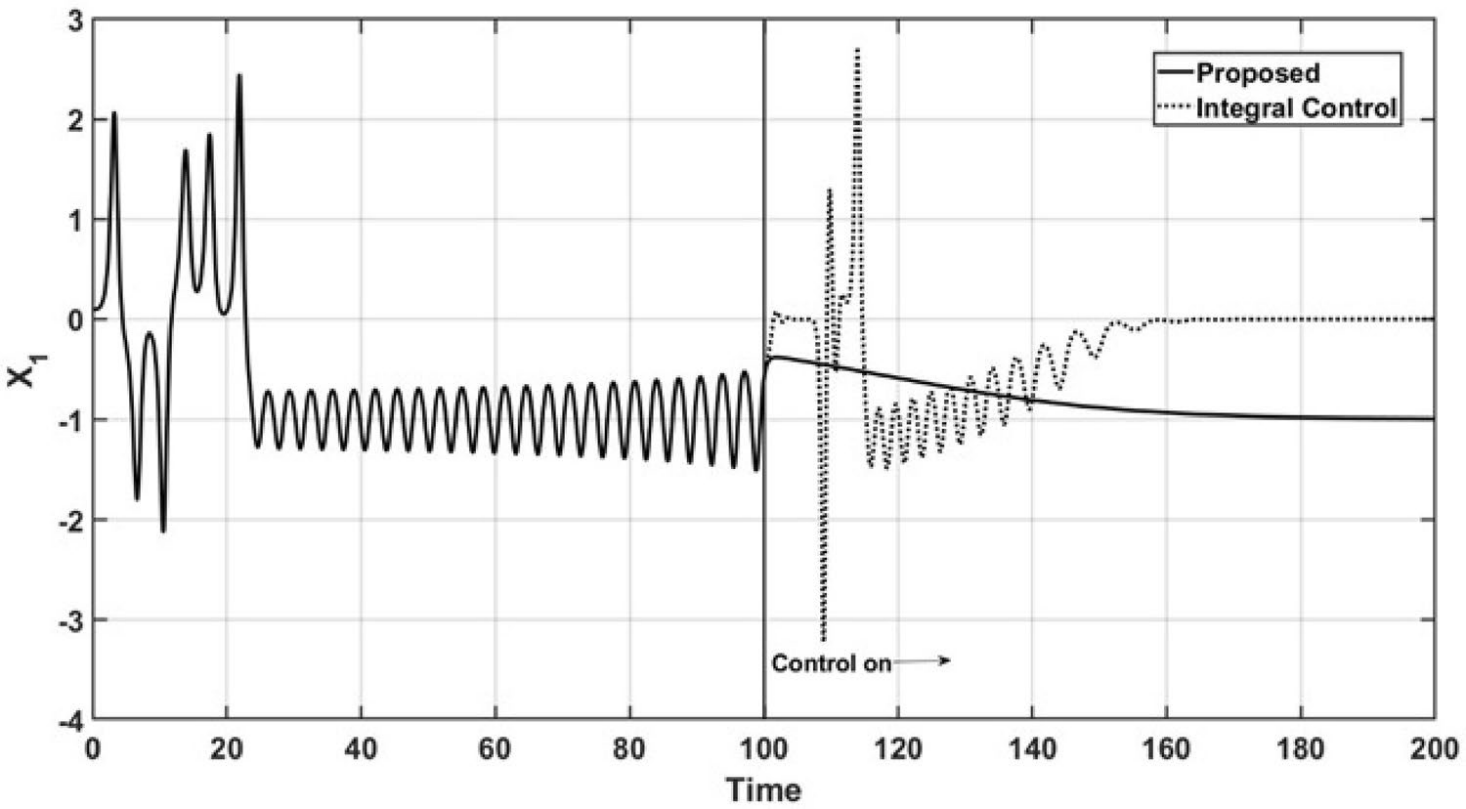
Trajectories of the uncontrolled variable $x_1$.

**Figure 3:**
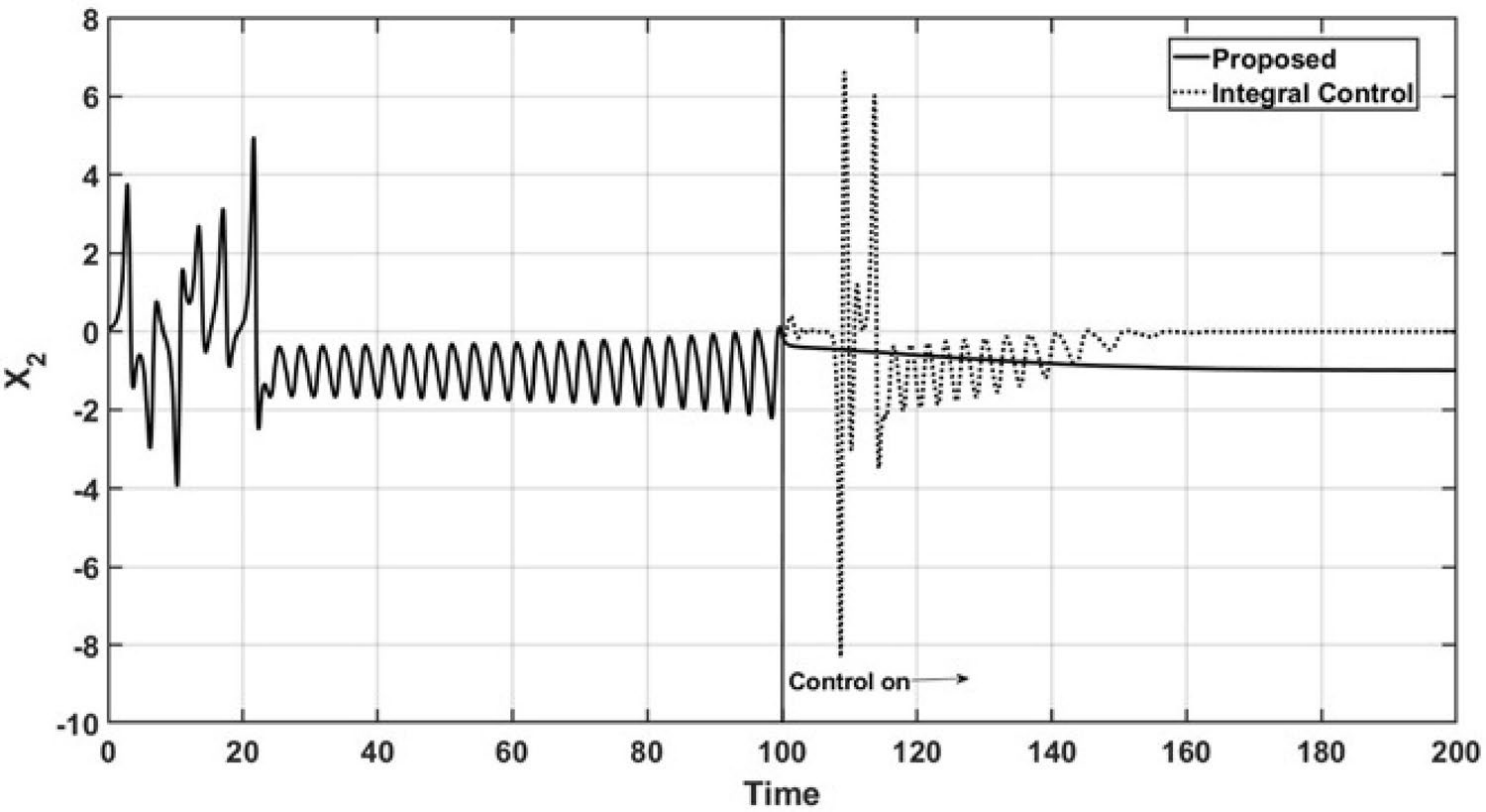
Trajectories of the uncontrolled variable $x_2$.

Figures 2 and 3 show the open-loop and closed-loop performance of the uncontrolled state variable trajectories, $x_1$ and $x_2$, respectively. As a consequence of the performance of the controlled state variable $x_3$, the oscillatory behavior is suppressed, and the trajectories are smoothly led to a steady state under the action of the proposed controller. Additionally, the trajectories of the uncontrolled state variables under the action of the integral controller maintain oscillatory behavior even after the control action is started, and it finally reaches a steady state.

**Figure 4:**
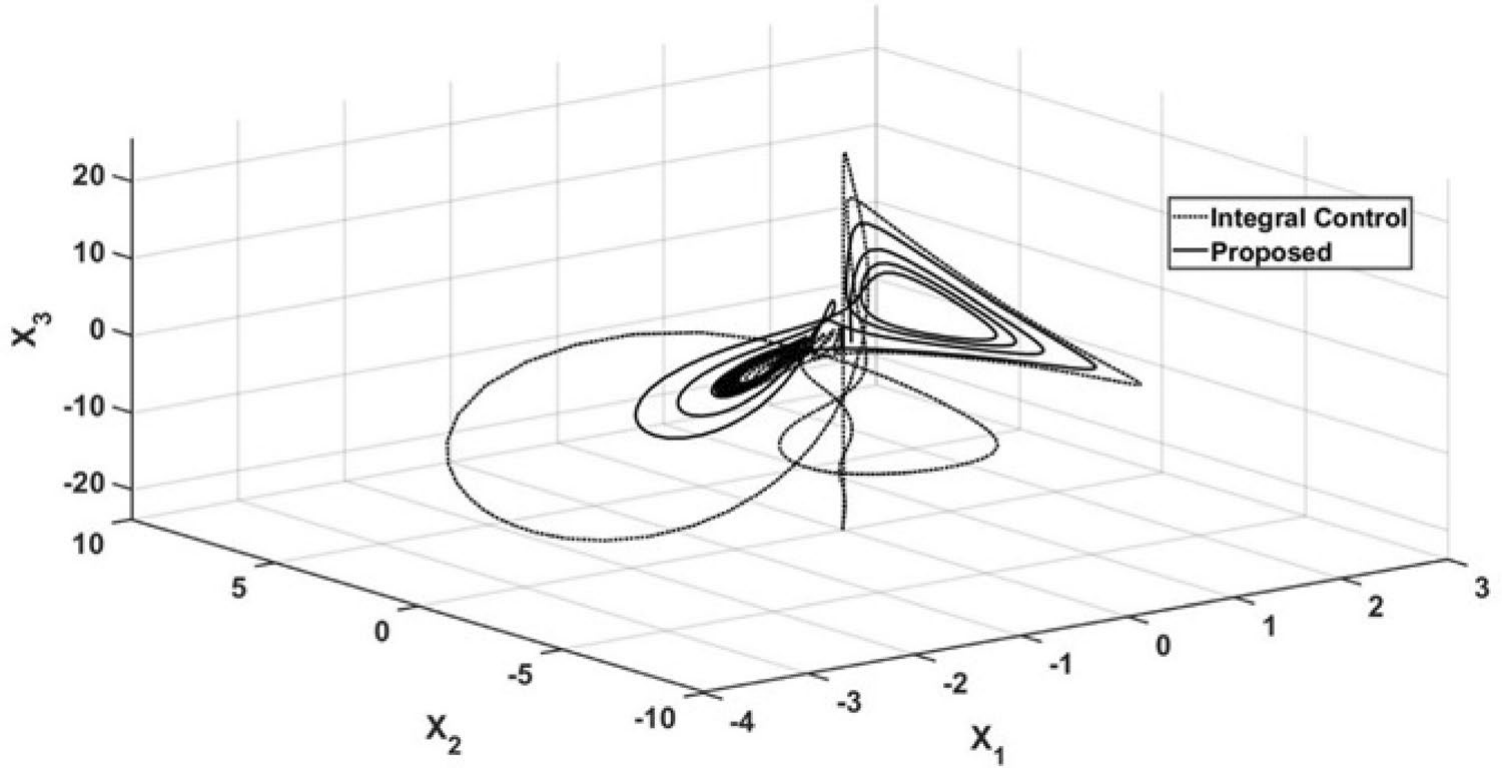
Phase portrait for regulation control.

The performance of the state variables is shown in Fig. 4, where a phase portrait is presented under the conditions mentioned in Figs. 1, 2 and 3. The corresponding orbit under the proposed controller arrives at the abovementioned steady state, with $x_3 = x_{3r}$ and the oscillatory behavior is suppressed. However, the corresponding orbit induced by the integral controller maintains oscillations with a wide ratio, and the orbit maintains oscillatory behavior.

**Figure 5:**
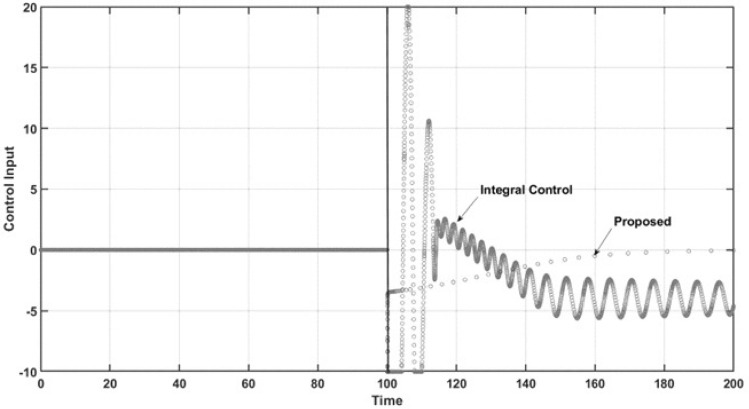
Control signals impacted by the regulation problem.

**Figure 6:**
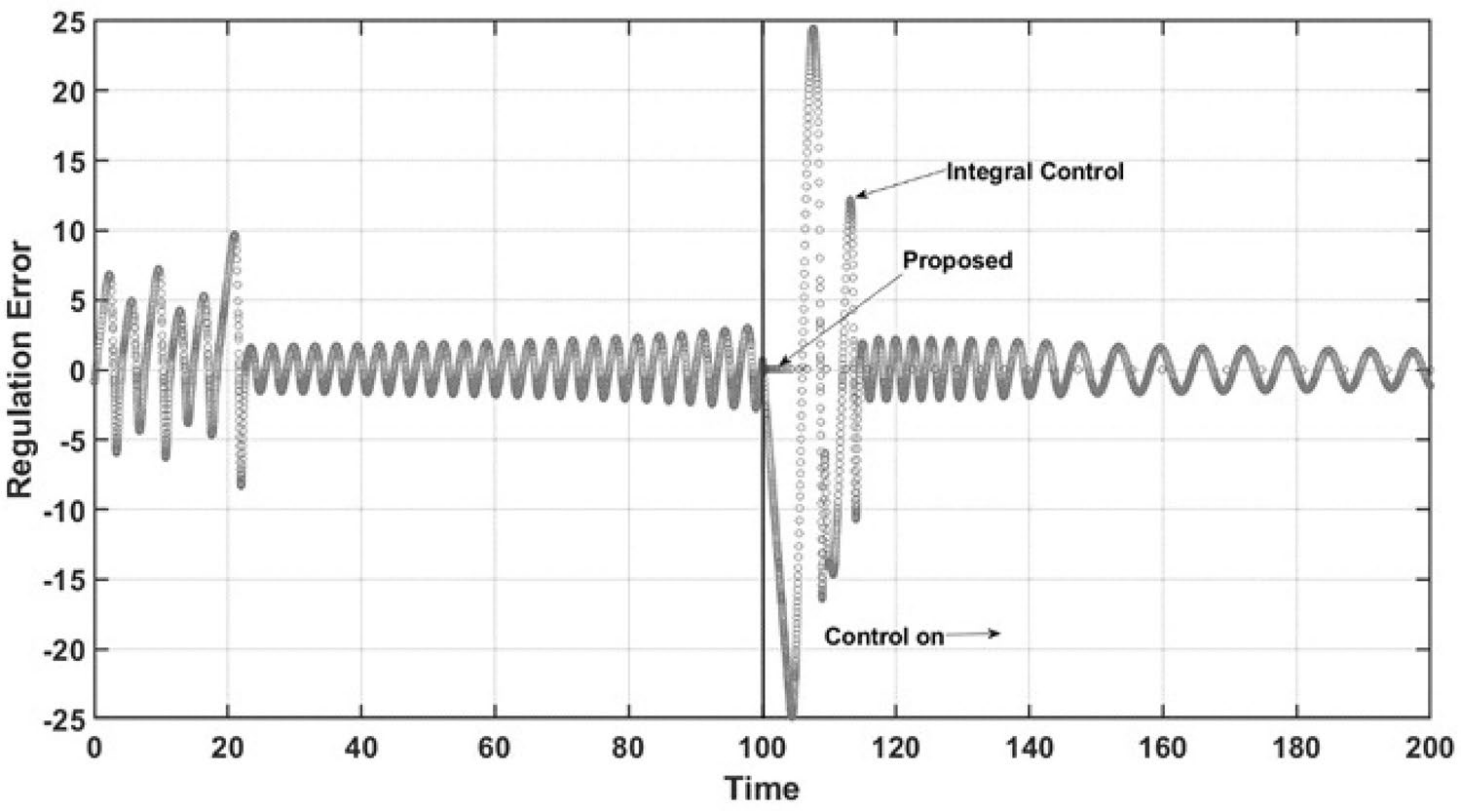
Regulation error.

The abovementioned behaviors of the state variables under both controllers can be explained by the performance of both controllers under comparison. Figure 5 demonstrates the control effort performance. The proposed controller has smooth behavior and practically does not reach saturation conditions. As expected, the controller has the desired anti-windup response, leading the trajectory of the controlled state variable to the required set point and preventing the oscillatory response of uncontrolled state variables, as mentioned above. The integral control law shows both lower and upper saturation while turning on, which is the named windup effect. It can be observed that the control effort is very high due to the large oscillation that occurs at the start of the closed-loop regimen and the sustained oscillation at steady-state conditions in practical applications. These characteristics are undesirable due to the potential of physical damage to the control actuator. Finally, for the regulation case, Fig. 6 shows the dynamic performance of the named regulation error *E*. When control occurs at $t = 100$ time units, the regulation error is zero when the proposed controller is turned on, which is in accordance with all the above results. For the integral control law, the expected oscillatory behavior is observed, which shows that the required set point is not reached.

**Figure 7:**
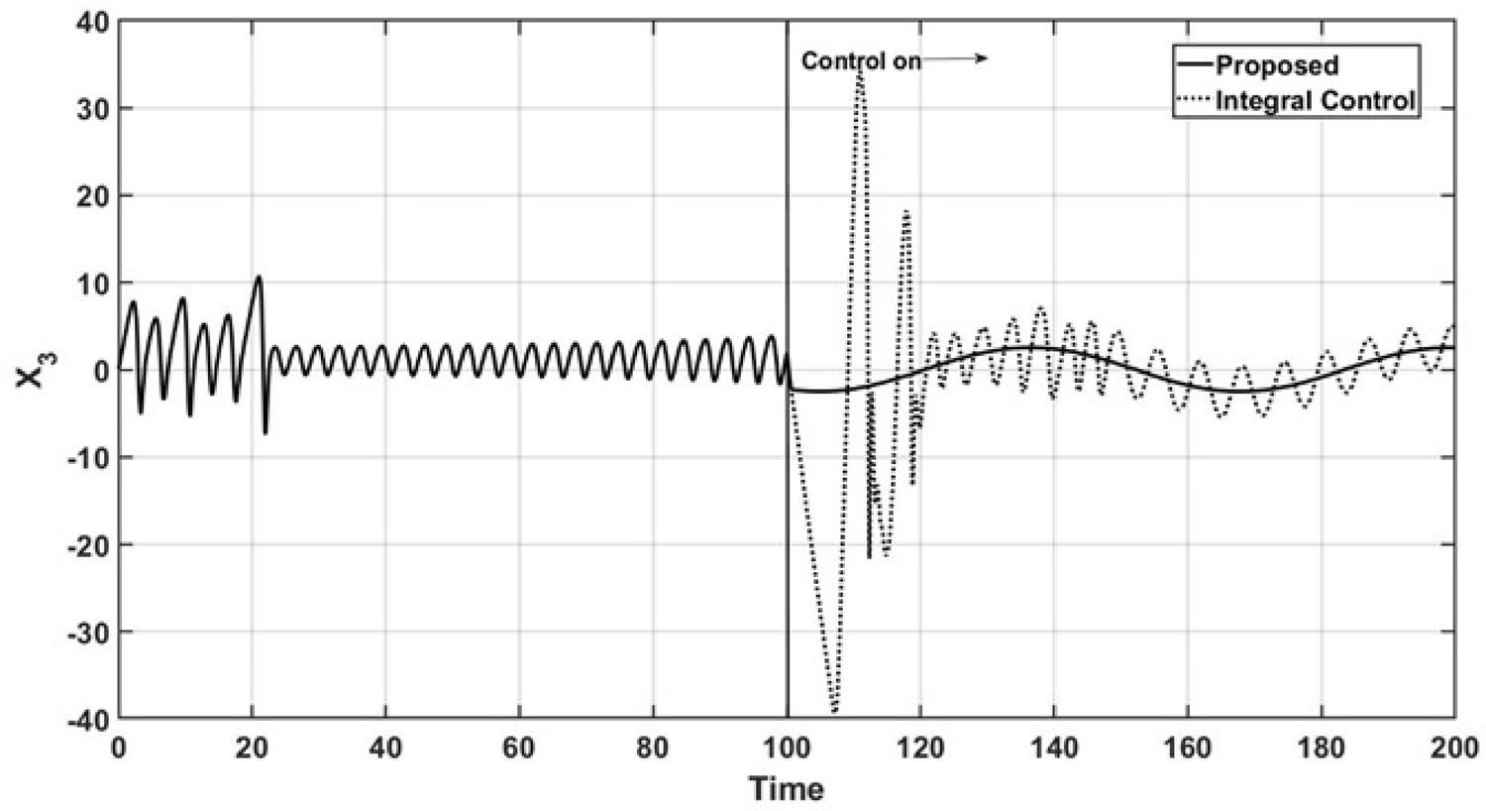
Tracking trajectory.

Now, the proposed controller is also able to force the controlled state variable to follow a specific sinusoidal trajectory, as previously described, changing the control objective to the tracking trajectory case. A similar set of numerical simulations was performed to show the performance of the proposed controller and the integral controller. Figure 7 shows the open-loop and closed-loop dynamic behavior of the controlled state variable $x_3$, and the controllers are turned on at $t = 100$ time units. The proposed controllers lead to the dynamic trajectory and almost instantaneously to the required sinusoidal trajectory without overshoots, and at the setting time, as observed the integral control law provokes high overshoots and the controller is not able to reach the required trajectory.

**Figure 8:**
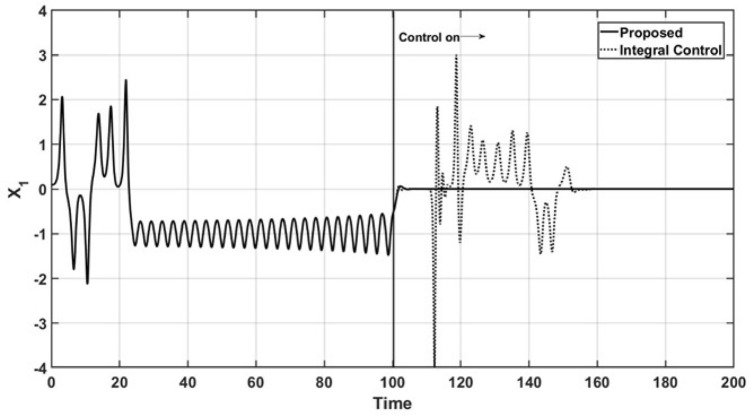
Behavior of the uncontrolled variable $x_1$.

**Figure 9:**
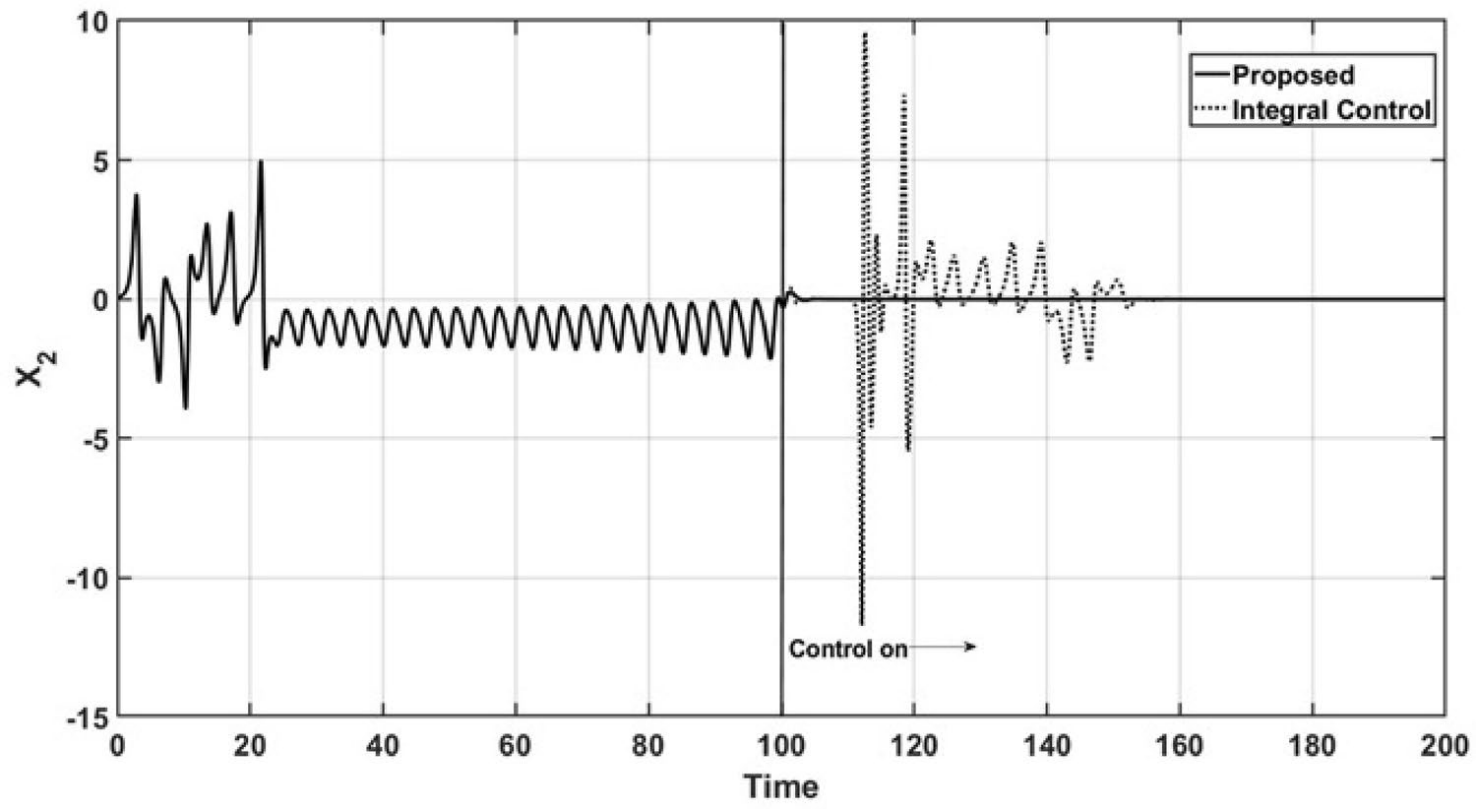
Behavior of the uncontrolled variable $x_2$.

Figures 8 and 9 show the dynamic behavior of the uncontrolled state variables, $x_1$ and $x_2$, in the tracking trajectory case. The sinusoidal behavior of the controlled state variable $x_3$ leads to the suppression of the complex oscillations of the uncontrolled state, where they reach a steady state faster.

**Figure 10:**
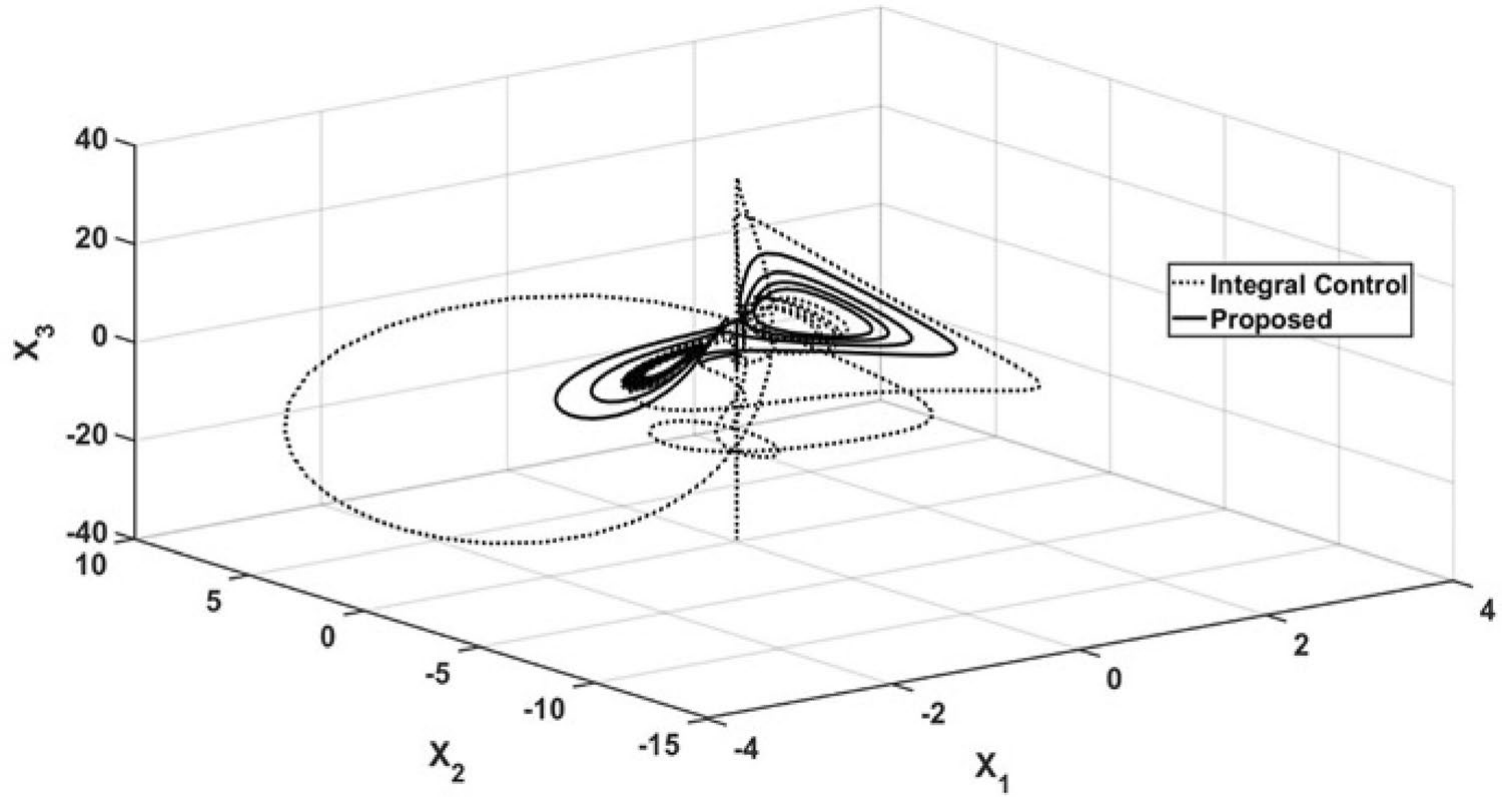
Phase portrait of the tracking trajectory.

As in the regulation case, a phase portrait of the tracking trajectory case is shown in Fig. 10. As in the above case, the wide orbit, which is related to the corresponding oscillatory behavior, is related to the action of the integral controller. This is different from the narrow orbit being forced by the action of the proposed control law, which forces the $x_3$ trajectory to reach the sinusoidal reference trajectory.

**Figure 11:**
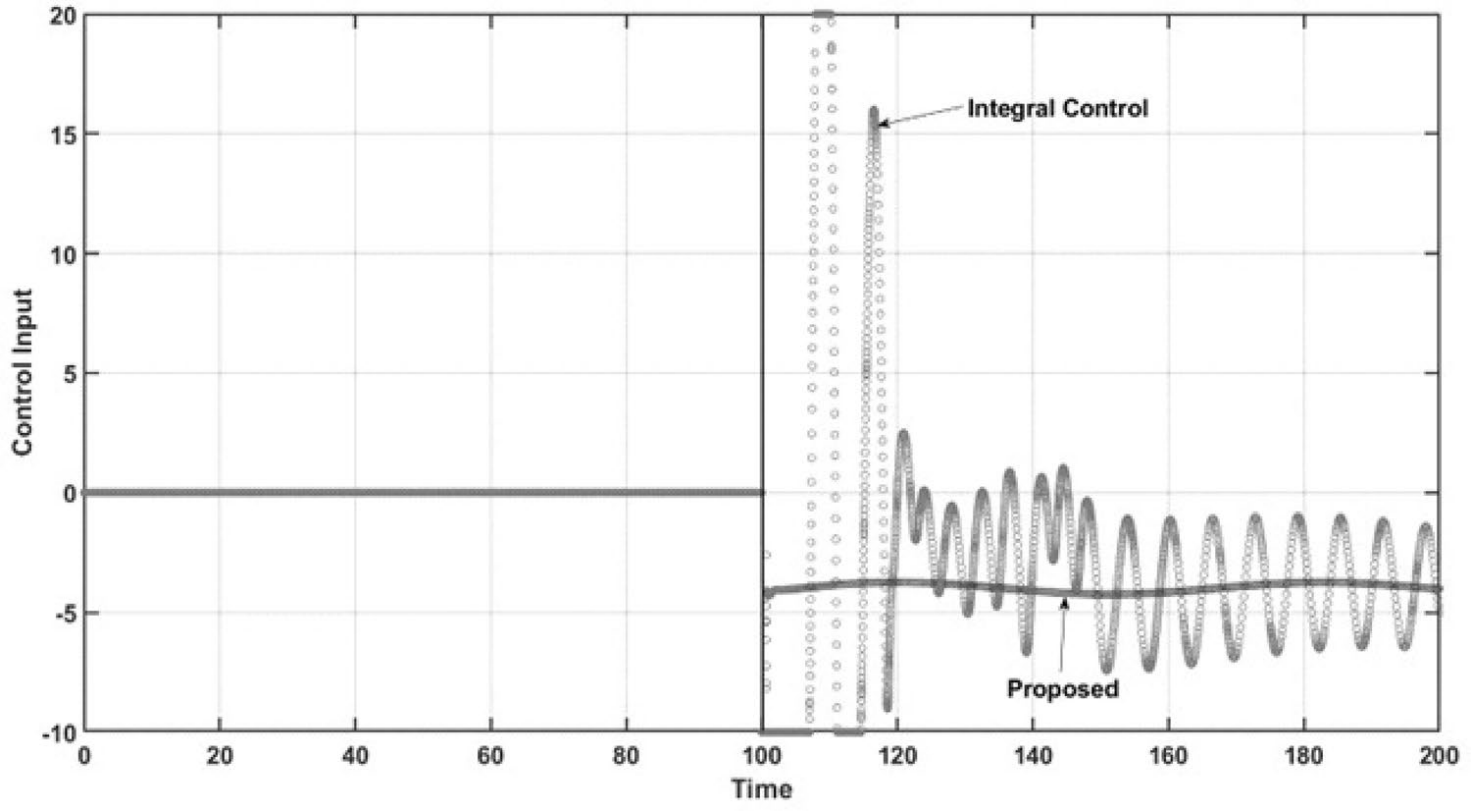
Control signals under the tracking trajectory.

**Figure 12:**
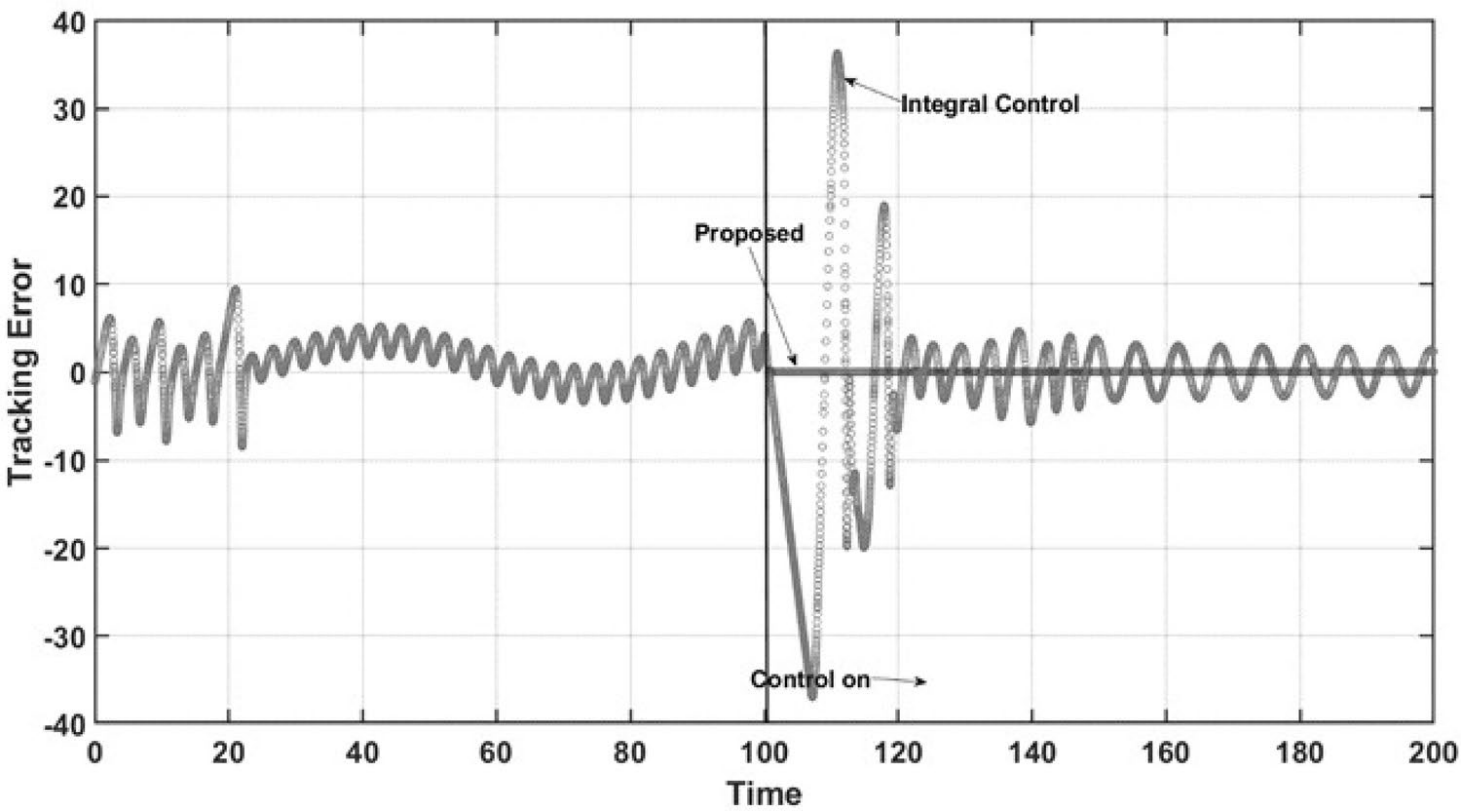
Tracking trajectory error.

Figure 11 is related to the performance of the control effort of both controllers. As can be observed, the integral control again suffers lower and upper saturation, making the controller unable to force the system to reach the reference trajectory and leading to high oscillations in the control effort, which is, as mentioned, undesirable. However, the proposed controller has an anti-windup effect, preventing the saturation phenomena, which allows the controller to force the required closed-loop objective well. Note that the proposed controller has a smooth oscillation, which is required to maintain the desired tracking trajectory. Finally, Fig. 12 shows the performance of the tracking error. Here, it is concluded that the proposed controller reaches its control objective adequately and without time delay, overshoots, or large setting times. Furthermore, the integral control law does not reach the desired trajectory, showing undesirable performance, with large oscillations.

## Conclusion

This work presents an alternative design for a class of integral controllers with adaptive gain. The adaptive gain is a function of the absolute values of the control error, where the main objective is to turn off the control action when the controller is saturated, thus preventing the named windup phenomena. The proposed methodology is successfully applied to a Rikitake-type chaotic oscillator for both regulation and tracking trajectory purposes such that the proposed control design can prevent the windup phenomena in the control saturation case. Numerical experiments show the performance of the considered methodology, and the proposed controller is compared with an equivalent integral controller with a fixed control gain.

## Data Availability

The datasets used and/or analyzed during the current study are available from the corresponding author upon reasonable request.
